# TaCD: Team-Aware Community Detection Based on Multi-View Modularity

**DOI:** 10.3390/e28010021

**Published:** 2025-12-24

**Authors:** Chengzhou Fu, Feiyi Tang, Lingzhi Hu, Chengzhe Yuan, Ronghua Lin

**Affiliations:** 1 College of Medical Information Engineering, Guangdong Pharmaceutical University, Guangzhou 510006, China; fucz@gdpu.edu.cn; 2 School of Information Engineering, Guangzhou Polytechnic University, Guangzhou 511483, China; tangfy@gzpyp.edu.cn; 3 School of Modern Information Industry, Guangzhou College of Commerce, Guangzhou 511363, China; 4 Faculty of Applied Sciences, Macao Polytechnic University, Macau 999078, China; 5 School of Electronics and Information, Guangdong Polytechnic Normal University, Guangzhou 510665, China; ycz@gpnu.edu.cn; 6 School of Computer Science, South China Normal University, Guangzhou 510631, China; rhlin@m.scnu.edu.cn

**Keywords:** team-aware, community detection, multi-view, Generalized Louvain, SCHOLAT

## Abstract

Community detection in social networks is one of the most important topics of network science. Researchers have developed numerous methods from various perspectives. However, the existing methods often overlook the team information encoded as a special type of user relation in the social network, which plays an important role in community formation and evolution. In this paper, we propose a novel community detection algorithm called Team-aware Community Detection (TaCD). Our model constructs a multi-view network by encoding the user interaction information as the user view and the team information as the team view. To measure the consistency across the two views, we use the Jaccard similarity to establish a cross-view coupling. Based on the constructed 2-view network, we use multi-view modularity to discover team-aware community structure, and solve the optimization problem using the well-known Generalized Louvain approach. Another contribution of this paper is the collection of a new SCHOLAT dataset, which consists of several social networks with team information and is publicly available for testing purposes. Our experimental results on several SCHOLAT networks with team information demonstrate that TaCD outperforms the existing community detection algorithms.

## 1. Introduction

Community detection is to discover both hidden and defined communities from the distributed and disordered structure of the internet and complex social systems [1,2,3]. Identifying communities can provide information about how the network is organized. It allows us to focus on areas of the graph that have a degree of autonomy. It also helps to classify vertices according to their roles relative to their communities [4]. Community detection has various applications in different fields, which can be used to uncover potential relationships between users in the field of social software development [5,6]. It can also be used to detect the structure of protein–protein interaction in the field of biology [7,8,9]. Even for the real internet, community detection can also be used to discover related websites [10,11,12,13]. In the era of big data, it is critical to discover the meaningful community structure when dealing with numerous huge networks.

**Figure 1:**
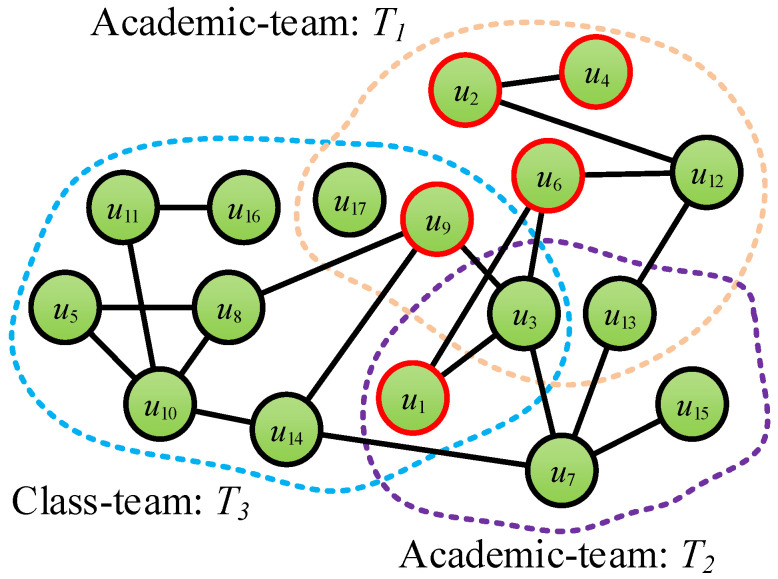
Illustration of network structure in SCHOLAT.

During the past few decades, more and more efforts have been made on community detection [14,15,16,17]. However, most of them only consider the user interaction or attribute information, but ignore the relationship with the teammates in the social network service platform. To demonstrate the importance of team information in community detection in social networks, we collect a dataset from SCHOLAT (SCHOLAT: https://www.scholat.com) [18] (a well-known academic social networking service platform in China). This dataset contains eight networks and will be made publicly available to all of the scholars for academic research usage. Different from the existing social network datasets, the social networks on the SCHOLAT dataset contain not only the user–user interaction information but also the team information. In SCHOLAT, the relationship between users is complex. There are many teams such as the Academic-team and Class-team which are created by users. As shown in Figure 1, there are three teams such as $T_{1}=\left\{u_{2},u_{3},u_{4},u_{6},u_{9},u_{12},u_{13},u_{17}\right\}$, $T_{2}=\left\{u_{1},u_{3},u_{7},u_{13},u_{15}\right\}$, and $T_{3}=\left\{u_{1},u_{3},u_{5},u_{8},u_{9},u_{10},u_{11},u_{14},u_{16},u_{17}\right\}$. Some users are in the same team, but they may not have friendship (e.g., in the team $T_{1}$: $u_{4}$ and $u_{9}$, $u_{2}$ and $u_{6}$, etc.). Similarly, the users have friendship, but they may not be in the same team (e.g., $u_{6}$ in the team $T_{1}$ and $u_{1}$ in the team $T_{2}$/$T_{3}$, etc.), and others may not be in the same team or have a friendship (e.g., $u_{4}$ in the team $T_{1}$ and $u_{1}$ in the team $T_{2}$ and $T_{3}$, etc.).

Unlike traditional multi-layer networks that typically aggregate homogeneous relationships (e.g., merging Twitter and Facebook ties), TaCD introduces a heterogeneous coupling mechanism. It integrates the explicit “Affiliation Layer” (Team-view) with the implicit “Interaction Layer” (User-view). By measuring the structural consistency between these two distinct views, we can effectively filter out noise where team membership does not reflect actual social proximity.

Despite the aforementioned importance of team information, it is mostly ignored in the existing methods. To this end, in this paper, we propose a new community detection method called Team-aware Community Detection (TaCD). In particular, a new 2-view network is constructed from the original network with team information, which consists of the user view for encoding user–user interaction and the team view for encoding team information. For measuring the consistency across the two views, the Jaccard similarity is adopted, whereby a cross-view coupling is established. Based on the newly constructed 2-view network, multi-view modularity is adopted to discover team-aware community structure, and solve this optimization problem using the well-known Generalized Louvain approach. Another contribution of this paper is that a new SCHOLAT dataset consisting of several social networks with team information is collected and made publicly available as a testing dataset. Extensive experiments are conducted to confirm the superiority of the proposed TaCD method over the existing methods.

The method introduced in this work has the following novel contributions: constructing a new dataset that is more suitable for large-scale networks, supporting multi-layer networks, and solving the coupling calculation problems between layers. The rest of this paper is organized as follows. We briefly review the related work in Section 2, and introduce the SCHOLAT dataset and research background in Section 3. In Section 4, the newly proposed Team-aware Community Detection approach is described in detail. In Section 5, extensive experiments are conducted to validate the effectiveness of the proposed method. At last, we will draw the conclusions and describe the future work in Section 6.

## 2. Related Work

In this section, we will briefly review some related work on community detection. One major type of community detection methods relies on designing a quality function, by solving the optimization problem of which the community structure can be discovered. For example, the normalized cuts (NCut)-based method measures both the total dissimilarity between the different groups as well as the total similarity within the groups, where a real valued solution to the normalized cut minimization problem is provided by a generalized eigenvalue system [19]. The non-negative matrix factorization (NMF) is an effective approach for community detection that utilizes a Bayesian model to extract overlapping modules from a network [20]. Cluster Affiliation Model for Big Networks (BIGCLAM) is another overlapping community detection method that scales to large networks of millions of nodes and edges. The method builds on a novel observation that overlaps between communities are densely connected [15]. Communities from Edge Structure and Node Attributes (CESNA) is an accurate and scalable algorithm for detecting overlapping communities in networks with node attributes. CESNA statistically models the interaction between the network structure and the node attributes, which leads to more accurate community detection as well as improved robustness in the presence of noise in the network structure [11]. Recently, some efforts have been made in higher-order community detection. It has been shown that higher-order features captured by network motifs are crucial in many domains, such as biology and neuron-science, which can help to gain new insights into the network organization beyond the clustering at the level of individual nodes and edges [21]. Rosvall and Bergstrom use the simulated annealing optimization algorithm and the effective coding of random walks for community detection [22]. Raghavan et al. propose a Label Propagation Algorithm (LPA), which is based on the idea that the edge of the network often represents the propagation of information [23]. A label update rule is further proposed for further reducing computational overhead [24]. Ma et al. [25] propose a novel algorithm by joint multi-label learning and feature extraction (MLjFE), where temporal link prediction and feature extraction are integrated into an overall objective function. Mahmood and Small present a community detection algorithm based on the fact that each network community spans a different subspace in the geodesic space. For making the process of community detection more robust, they use sparse linear coding with $\ell_{1}$ norm constraint [26]. Jin proposes an approach to community detection termed Spectral Clustering On Ratios-of-Eigenvectors (SCORE), the main innovation of which is to use the entry-wise ratios between the first leading eigenvector and each of the other leading eigenvectors for clustering [27]. In fact, in community detection, new data are always generated continuously with subgraphs joining simultaneously in dynamic evolving networks. For addressing the above problem, Zhao et al. present a method to detect communities by handling subgraphs [28].

Recently, multi-view community detection has been widely studied, such as modularity-based methods [29,30] and information-theoretic methods [31]. Modularity evaluates the quality of a network partition, where a higher modularity value usually indicates a denser edge distribution within communities [32,33]. Furthermore, Delvenne et al. derive the multi-layer modularity by assessing the capability of the given community structure to capture a dynamic process in a multi-layer network [34]. Ma et al. propose a quantitative function (multi-layer modularity density), while considering the connection information among various layers [35] for community detection in multi-layer networks.

**Figure 2:**
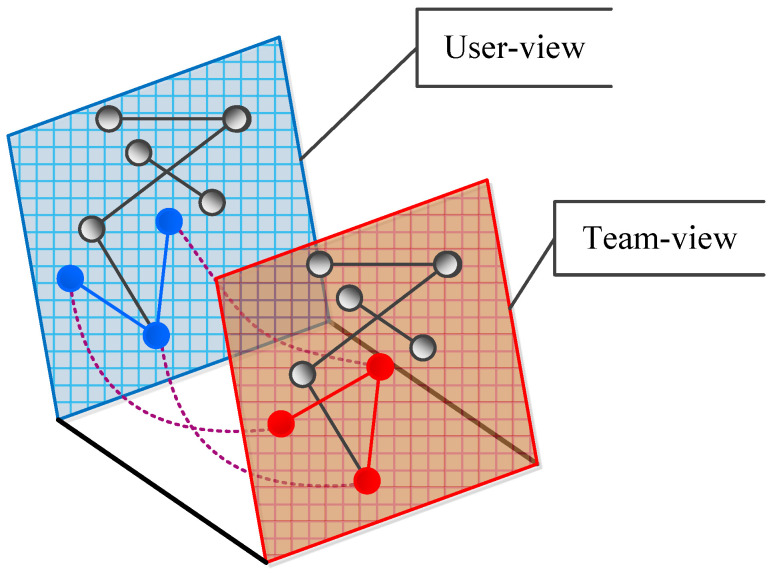
The User-view (blue) and Team-view (red) in our method. Solid lines represent interaction intensity, while dashed lines represent coupling.

However, the algorithms described above ignore team information. The purpose of this paper is to enhance the performance of community detection by incorporating team information from the datasets. In particular, we use the multi-view method to detect team-aware communities as shown in Figure 2. For example, with the three blue nodes in User-view and three red nodes in Team-view, although they represent the same three nodes, they have different relationships in the two views.

Recent advancements have also explored dynamic and feature-based clustering. For instance, matrix factorization has been revisited for dynamic graph clustering [36], and joint learning frameworks have been proposed for large-scale temporal networks [37]. While effective, our approach differs by focusing specifically on leveraging static affiliation metadata as a structural view.

## 3. Dataset Description

In this section, we describe the SCHOLAT dataset from the following perspectives.

### 3.1. About SCHOLAT and Its Dataset

SCHOLAT is an academic social networking service website designed to promote exchanges and cooperation between researchers. The platform contains multiple features such as academic information management, literature search, academic network disk, teaching course management, and scholar exchange services.

Since its establishment, SCHOLAT has gained widespread recognition and has attracted a large number of scholars, teachers, and students to utilize its services. It serves as a scientific research platform for scholars, focusing on engineering applications, theoretical research, and academic exchange. SCHOLAT assists scientific researchers in building their own academic networks, helps students find suitable mentors, and provides up-to-date job opportunities for those seeking scientific research positions.

**Figure 3:**
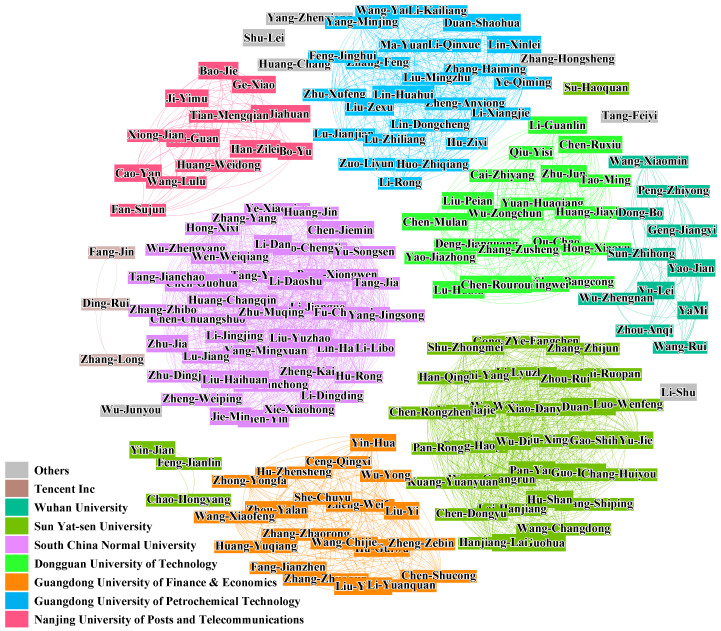
The real community relationship in the SCHOLAT dataset: we see that only the nodes inside the community have a relationship, and there is no connection between the nodes in different communities.

A given sample of real community relationship in SCHOLAT is shown in Figure 3, and a community summary is shown in at bottom left of the figure.

It is important to note that we cannot directly regard team as a community, because the concept of team is only relative to the users of SCHOLAT platform, but the underlying community is the collective division in real life.

For users, their relationship at the User-view and at the Team-view level are independent, but if we make good use of the relationship between the two levels in community detection, it can help us to have a better understanding of the relationship between users.

To this end, the motivation of this research can be addressed as it is necessary to consider team information on community detection. To tackle this issue, we propose a novel method for Team-aware Community Detection in social networks.

### 3.2. The Networks in Dataset

As shown in Table 1, we use the eight networks to do the experiments, namely, Net-3k, Net-4k, …, and Net-10k. The Nk notation (e.g., Net-3k) denotes a network with n=N×1000 nodes (e.g., n=3000). Specifically, the largest network consists of 10,000 nodes (with 5,713,566 edges and 218 communities). The work departments of users on the dataset mainly contain universities, academic organizations, and companies from China. In addition, these users are almost all registered with real names, and the quality of their information is also generally high. To protect the users’ privacy, we discretize their ids (If you use this dataset in your work, please cite this publication. You can download the dataset and related code from here (the password of the zip file is “*Goodluck!*”): https://www.scholat.com/research/tacd, accessed on 13 November 2025).

The SCHOLAT dataset includes the following files, which are

- *user_real_community.csv*: the row number represents user id, and value in each cell is the real community id;
- *link_friendship.csv*: the link information of friendship as $\langle user_{1},user_{2}\rangle$, using user follower/followee relations;
- *matrix_common_team_count.csv*: contains three columns such as $user_{1}$, $user_{2}$ and the number of common teams;
- *matrix_interact_times.csv*: which contains interaction information, constructed via Equation (1) in Section 4.1;
- *matrix_friendship.csv*: the matrix of *link_friendship.csv*;
- *matrix_jaccard.csv*: which computed via Equation (4) in Section 4.2.

**Example** **1.** *In the net-3k with 3000 nodes and 689,480 edges, the user id range is from 1 to 3000. There are 157 communities in the real world, including Apple Inc., DIGITO Agency, Faimdata, Tai Fung Bank, Guangdong Pharmaceutical University, China University of Geosciences (Beijing) and South China Normal University et al. The users’ names are those such as Yong-Tang, Na-Tang, Xiao-Liu, Long-Zhang, Yuncheng-Jiang, and Li-Huang et al.*

## 4. The Proposed TaCD Method

In TaCD, we propose a Generic Multi-view Interaction Framework designed to bridge heterogeneous social information. Unlike traditional multi-layer networks that aggregate homogeneous ties, our framework constructs a network consisting of two distinct logical views: an Interaction Layer (View *s*) representing implicit pairwise behaviors, and an Affiliation Layer (View *r*) representing explicit shared group memberships.

### 4.1. Constructing Matrices of the View Information

The conceptual structure is illustrated in Figure 2. For example, the three blue nodes in the User-view (Interaction Layer) and the three red nodes in the Team-view (Affiliation Layer) represent the same entities but exhibit different topological relationships. Our method exploits this complementary information.

The proposed framework is formalized by a set of adjacency matrices ${𝒜}_{\left(i,j\right)}^{\left[*\right]}$, specifically comprising the Interaction View ${𝒜}_{\left(i,j\right)}^{\left[s\right]}$ and the Affiliation View ${𝒜}_{\left(i,j\right)}^{\left[r\right]}$. Here, ${𝒜}_{\left(i,j\right)}^{\left[s\right]}$ is the adjacency matrix encoding the frequency of interactions between user pairs, and ${𝒜}_{\left(i,j\right)}^{\left[r\right]}$ represents the matrix quantifying the extent of shared team affiliations. The detailed construction of these matrices is described as follows.

(1) Interaction Layer (View *s*): This layer encodes the intensity of pairwise user interactions. In a general social network, this is formalized as a weighted sum of various interaction types. Taking the SCHOLAT dataset as a specific case study, we instantiate this layer by mapping *Friendship*, *Like*, and *Chat* behaviors to the interaction weights:

(1) $${𝒜}_{\left(i,j\right)}^{\left[s\right]}=\chi F\left(i,j\right)+\psi L\left(i,j\right)+\omega C\left(i,j\right)$$

We can compute the *Friendship* value F(i,j) of user *i* and *j* as

(2) $$F\left(i,j\right)=\left\{\begin{array}{ccc} 1 & , & i\;\mathrm{and}\;j\;\mathrm{are}\;\mathrm{friends}\;\\ 0 & , & \;\mathrm{otherwise}\; \end{array}\right.$$

where F(i,j) is the *Friendship* value of user *i* and *j* are computed via Equation (2). The L(i,j) represents the number of times user *i* performs a ‘Like’ action on user *j* (or vice versa). The C(i,j) represents the frequency with which user *i* and *j Chat* with each other.

In our proposed generic framework, the weighting parameters χ, ψ, and ω are tunable to reflect the varying importance of different interaction types across specific platforms. In the context of the SCHOLAT dataset, empirical observations suggest that explicit social ties imply stronger connections than casual interactions. Therefore, we set χ=2 to emphasize the importance of friendship, while setting ψ=1 and ω=1 for ‘Like’ and ‘Chat’ interactions, respectively.

Intuitively, the relationships among nodes are more important than other types of interaction, so it should have greater weight. Therefore, in this paper, we let χ=2 and ψ=ω=1.

**Figure 4:**
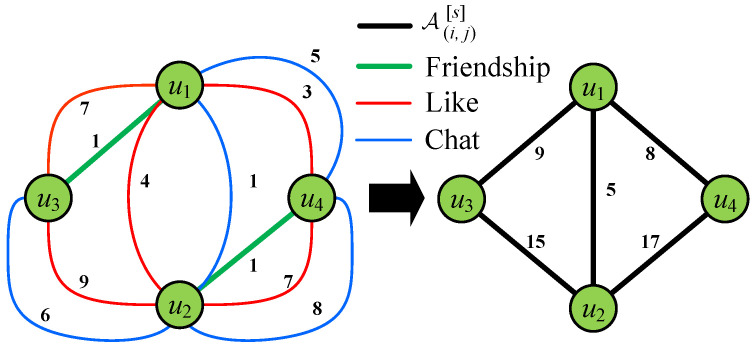
Illustration of interaction among $u_{1},u_{2},u_{3},u_{4}$: there is no interaction of any type between $u_{3}$ and $u_{4}$, so there is no connection between them (the weight of edges is zero).

According to Equation (1), consider a scenario, where SCHOLAT users $u_{1},u_{2},u_{3},u_{4}$ interact using the public interactions in view *s* as shown in Table 2 and Figure 4.

**Example** **2.** *The ${𝒜}_{\left(i,j\right)}^{\left[s\right]}$ of user $u_{2}$ and $u_{4}$ in the interaction is computed as ${𝒜}_{\left(u_{2},u_{4}\right)}^{\left[s\right]}=2\times 1+1\times 7+1\times 8=17$.*

**Figure 5:**
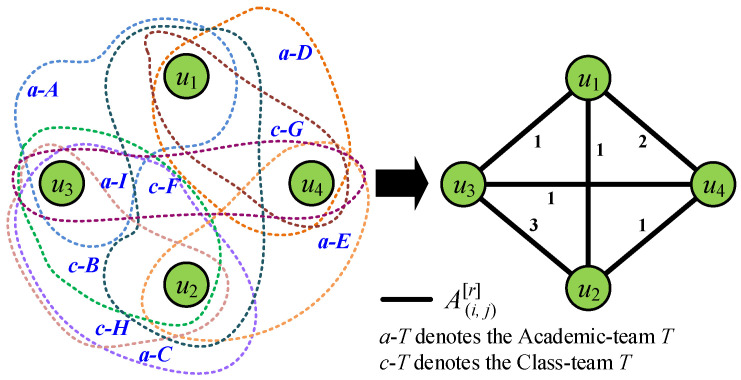
Illustration of common teams among $u_{1},u_{2},u_{3},u_{4}$: the number of common teams between nodes constitutes their connection at the Team-view.

(2) Number of common teams (View *r*): This layer represents shared group memberships. ${𝒜}_{\left(i,j\right)}^{\left[r\right]}$ quantifies explicit groups shared by users. Taking Academic-teams and Class-teams as specific instances in our case study, the affiliation matrix is defined as

(3) $${𝒜}_{\left(i,j\right)}^{\left[r\right]}=M\left(i,j\right)+N\left(i,j\right)$$

where M(i,j) denotes the number of common Academic-teams and N(i,j) denotes the number of common Class-teams in SCHOLAT of user *i* and *j*. We now consider the second scenario, where SCHOLAT users $u_{1},u_{2},u_{3},u_{4}$ denote common team-ship using the public team member information in view *r* as shown in Table 3 and Figure 5.

**Example** **3.** *The user $u_{2}$ and $u_{3}$ have the one common Academic-team a-C, and two Class-teams such as c-B and c-H, so ${𝒜}_{\left(i,j\right)}^{\left[r\right]}$ of user $u_{2}$ and $u_{3}$ in view r is computed as ${𝒜}_{\left(u_{2},u_{3}\right)}^{\left[r\right]}=1+2=3$.*

### 4.2. Constructing a Matrix of Interaction Between Multi-View

The motivation for using Jaccard similarity is to act as a structural consistency filter. In social networks, a “team” relationship might exist without actual social interaction (noise). By computing the overlap of neighbors between the interaction view and the team view, we assign a high coupling strength only when the team structure aligns with the social structure. In this section, we use Jaccard similarity between view *s* and *r* to construct a matrix, which measures the cross-view clustering consistency. We need to define the data consistency for the two views as follows: (1) For the view *s*, let $\Gamma_{\left(i\right)}^{\left[s\right]}$ denote the friends of user *i*. (2) For the view *r*, let $\Gamma_{\left(j\right)}^{\left[r\right]}$ denote the teammates of user *j*, to this end, $J_{\left(i,j\right)}^{\left[sr\right]}$ can be computed as

(4) $$\begin{array}{c} J_{\left(i,j\right)}^{\left[sr\right]}=Jaccard\left(\Gamma_{\left(i\right)}^{\left[s\right]},\Gamma_{\left(j\right)}^{\left[r\right]}\right)=\frac{|\Gamma_{\left(i\right)}^{\left[s\right]}\cap \Gamma_{\left(j\right)}^{\left[r\right]}|}{|\Gamma_{\left(i\right)}^{\left[s\right]}\cup \Gamma_{\left(j\right)}^{\left[r\right]}|} \end{array}$$

In this paper, we only measure the cross-view clustering consistency for each user, so we set i=j in Equation (4). Given a user *u*, the Jaccard similarity between the view *s* and *r* for user *u* can be computed as

(5) $$\begin{array}{c} J^{\left[sr\right]}\left(u\right)=J_{\left(u,u\right)}^{\left[sr\right]}\;=Jaccard(\Gamma_{\left(u\right)}^{\left[s\right]},\Gamma_{\left(u\right)}^{\left[r\right]})=\frac{|\Gamma_{\left(u\right)}^{\left[s\right]}\cap \Gamma_{\left(u\right)}^{\left[r\right]}|}{|\Gamma_{\left(u\right)}^{\left[s\right]}\cup \Gamma_{\left(u\right)}^{\left[r\right]}|} \end{array}$$

**Figure 6:**
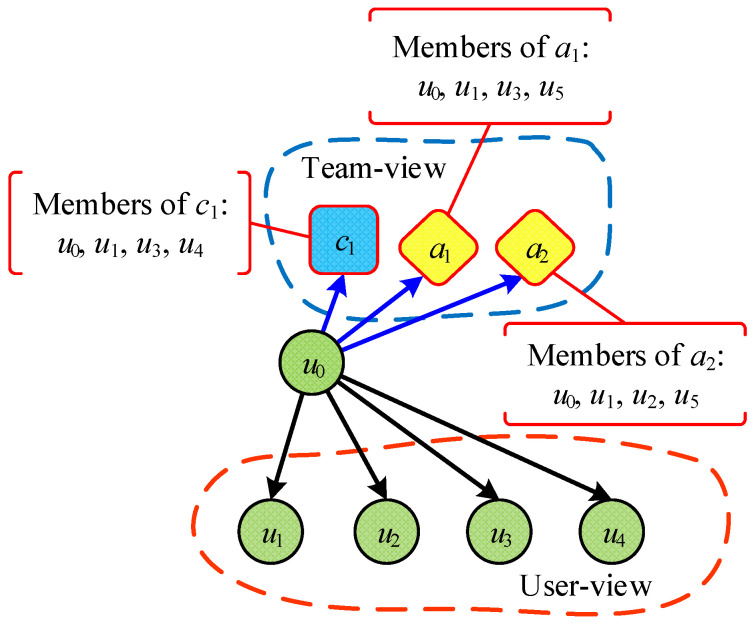
Illustration of data view consistency: Data view consistency across view *s* and view *r* of user $u_{0}$: $J^{\left[sr\right]}\left(u_{0}\right)=\frac{|\left\{u_{1},u_{2},u_{3},u_{4}\right\}\cap \left\{u_{1},u_{2},u_{3},u_{4},u_{5}\right\}|}{|\left\{u_{1},u_{2},u_{3},u_{4}\right\}\cup \left\{u_{1},u_{2},u_{3},u_{4},u_{5}\right\}|}=0.8$.

**Example** **4.** *We can use the number of the common friends to measure the similarity between the User-view and Team-view. According to Figure 6, we can find (1) tn the User-view: the friends of user $u_{0}$ are $\left\{u_{1},u_{2},u_{3},u_{4}\right\}$. (2) In the Team-view: the user $u_{0}$ has the team $a_{1}$, $a_{2}$, and $c_{1}$ (the nodes with red border, where a denotes Academic-team, and c denotes Class-team), while the other members are $\left\{u_{1},u_{3},u_{4}\right\}$, $\left\{u_{1},u_{3},u_{5}\right\}$, and $\left\{u_{1},u_{2},u_{5}\right\}$. The friends of user $u_{0}$ are $\left\{u_{1},u_{3},u_{4}\right\}\cup \left\{u_{1},u_{3},u_{5}\right\}\cup \left\{u_{1},u_{2},u_{5}\right\}=\left\{u_{1},u_{2},u_{3},u_{4},u_{5}\right\}$. In the end, we can compute the Jaccard similarity between view s and r of user $u_{0}$ as $J^{\left[sr\right]}\left(u_{0}\right)=J_{\left(u_{0},u_{0}\right)}^{\left[sr\right]}=\frac{|\left\{u_{1},u_{2},u_{3},u_{4}\right\}\cap \left\{u_{1},u_{2},u_{3},u_{4},u_{5}\right\}|}{|\left\{u_{1},u_{2},u_{3},u_{4}\right\}\cup \left\{u_{1},u_{2},u_{3},u_{4},u_{5}\right\}|}=0.8$. The technical details for constructing matrix are as shown in Algorithm 1.*

**Algorithm 1** The Coupling Matrix Construction Algorithm**Input:** *n* denotes the number of nodes, and the neighbor sets in the user-view $\Gamma_{\left(i\right)}^{\left[s\right]}$ and team-view $\Gamma_{\left(i\right)}^{\left[r\right]}$ for all i∈[1,n].**Output:** The diagonal coupling matrix $J^{\left[sr\right]}\in R^{n\times n}$.
 1:Initialize $J^{\left[sr\right]}$ as an n×n zero matrix. 2:**for** i=1 to *n* **do** 3:  Compute $J^{\left[sr\right]}\left(i\right)=\frac{|\Gamma_{\left(i\right)}^{\left[s\right]}\cap \Gamma_{\left(i\right)}^{\left[r\right]}|}{|\Gamma_{\left(i\right)}^{\left[s\right]}\cup \Gamma_{\left(i\right)}^{\left[r\right]}|}$                          ▹Using Equation (5) 4:

$J_{\left(i,i\right)}^{\left[sr\right]}\leftarrow J^{\left[sr\right]}\left(i\right)$

 5:
**end for**
 6:**return** $J^{\left[sr\right]}$


### 4.3. The TaCD Method

There is currently no recognized community definition, which is only qualitatively considered to be a set of vertices with a “tight inside and loose outside” structure. In order to quantify the “tightness and looseness”, Newman and Girvan [32] propose the modularity $Q_{M}\in \left(0,1\right)$, which is the most popular quality function for community detection. The modularity $Q_{M}$ can be defined as

(6) $$\begin{array}{c} Q_{M}=\frac{1}{2m^{\left[*\right]}}\sum_{\left(i,j\right)}\left[{𝒜}_{\left(i,j\right)}^{\left[*\right]}-\frac{k_{i}k_{j}}{2m^{\left[*\right]}}\right]\delta \left(g_{i},g_{j}\right)\;=\frac{1}{2m^{\left[*\right]}}\left[\sum_{\left(i,j\right)}{𝒜}_{\left(i,j\right)}^{\left[*\right]}-\frac{\sum_{i}k_{i}\sum_{j}k_{j}}{2m^{\left[*\right]}}\right]\delta \left(g_{i},g_{j}\right) \end{array}$$

where ${𝒜}_{\left(i,j\right)}^{\left[*\right]}$ represents the weight of the edge between node *i* and *j*. $k_{i}=\sum_{j}{𝒜}_{\left(i,j\right)}^{\left[*\right]}$ is the sum of the weights of the edges attached to vertex *i*. $g_{i}$ is the community to which vertex *i* is assigned, and δ(x,y) is the Kronecker delta [38], e.g., δ(x,y)=1 if x=y, otherwise δ(x,y)=0. $m^{\left[*\right]}=\frac{1}{2}\sum_{\left(i,j\right)}{𝒜}_{\left(i,j\right)}^{\left[*\right]}$ denotes the number of inner edges of an adjacent matrix [30].

Part of the algorithm efficiency results from the fact that the gain in modularity $\Delta Q_{M}$ [2] obtained by moving an isolated node *i* into a given community *C* can easily be computed as

(7) $$\begin{array}{c} \Delta Q_{M}=\left[\frac{\sum_{in}+k_{\left(i,in\right)}}{2m^{\left[*\right]}}-{\left(\frac{\sum_{tot}+k_{i}}{2m^{\left[*\right]}}\right)}^{2}\right]-\;\left[\frac{\sum_{in}}{2m^{\left[*\right]}}-{\left(\frac{\sum_{tot}}{2m^{\left[*\right]}}\right)}^{2}-{\left(\frac{k_{i}}{2m^{\left[*\right]}}\right)}^{2}\right] \end{array}$$

where

- $\sum_{in}$: the sum of the weights of the links inside *C*;
- $\sum_{tot}$: the sum of the weights of the links incident to nodes in *C*;
- $k_{i}$: the sum of the weights of the links incident to node *i*;
- $k_{i,in}$: the sum of the weights of the links from *i* to nodes in *C*;
- $m^{\left[*\right]}$: the sum of the weights of all the links on the network.

The objective function of modularity can be computed using 𝒜 and C as input. To solve this, the Generalized Louvain algorithm [39] can be used. For a more detailed explanation of its implementation, please refer to [40].

Using the steady-state probability distribution $P_{j}^{[r]*}=\frac{\kappa_{j}^{\left[r\right]}}{2\mu }$, where $2\mu =\sum_{j}^{\left[r\right]}\kappa_{\left(i,j\right)}$, we obtain the multi-view null model in terms of the probability $\rho_{i}^{\left[s\right]}{|}_{j}^{\left[r\right]}$ of sampling node *i* in view *s* conditional on whether the multi-view structure allows one to step from (j,r) to (i,s), accounting for view *s* and *r* steps separately [30] as

(8) $$\begin{array}{c} \rho_{i}^{\left[s\right]}{|}_{j}^{\left[r\right]}P_{j}^{[r]*}=\frac{1}{2\mu }\left(\frac{k_{i}^{\left[s\right]}}{2m^{\left[s\right]}}\frac{k_{j}^{\left[r\right]}}{\kappa_{j}^{\left[r\right]}}\delta \left(s,r\right)+\frac{C_{j}^{\left[sr\right]}}{c_{j}^{\left[r\right]}}\frac{c_{j}^{\left[r\right]}}{\kappa_{j}^{\left[r\right]}}\delta \left(i,j\right)\right)\kappa_{j}^{\left[r\right]} \end{array}$$

(9) $$\begin{array}{cc} Q^{\left[sr\right]}= & \frac{1}{2\mu }\sum_{\left(i,j\right)}^{\left[sr\right]}\left[\left({𝒜}_{\left(i,j\right)}^{\left[s\right]}-\gamma_{v}\frac{k_{i}^{\left[s\right]}k_{j}^{\left[s\right]}}{2m^{\left[s\right]}}\right)\delta \left(s,r\right)+\;C_{j}^{\left[sr\right]}\delta \left(i,j\right)\right]\delta \left(g_{i}^{\left[s\right]},g_{j}^{\left[r\right]}\right) \end{array}$$

where each network view *s* is represented by an adjacency ${𝒜}_{\left(i,j\right)}^{\left[s\right]}$ between node *i* and *j*, with view couplings $C_{j}^{\left[sr\right]}$ that connect node *j* in view *r* to itself in view *s*.

In TaCD, we use the Jaccard similarity to compute the coupling of view *s* and view *r*, combining with Equation (4), our proposed method to compute the modularity as

(10) $$\begin{array}{ccc} \tilde{Q}\left(g_{i}^{\left[s\right]}\right)=\; & \frac{1}{2\mu }\sum^{\left[sr\right]}\sum_{\left(i,j\right)}[\left({𝒜}_{\left(i,j\right)}^{\left[s\right]}-\gamma_{v}\frac{k_{i}^{\left[s\right]}k_{j}^{\left[s\right]}}{2m^{\left[s\right]}}\right)\delta \left(s,r\right)+\; & J_{\left(i,j\right)}^{\left[sr\right]}\delta \left(i,j\right)]\delta (g_{i}^{\left[s\right]},g_{j}^{\left[r\right]}) \end{array}$$

where $J_{\left(i,j\right)}^{\left[sr\right]}$ can be computed via Equation (4). ${𝒜}_{\left(i,j\right)}^{\left[s\right]}$ denotes the matrix of view *s*. $g_{i}^{\left[s\right]}$ denotes the community label of node *i* in view *s*. δ(x,y) is the Kronecker delta, which is the same as Equation (6). $k_{i}^{\left[s\right]}=\sum_{j=1}^{n}{𝒜}_{\left(i,j\right)}^{\left[s\right]}$ and $m^{\left[s\right]}=\sum_{i=1}^{n}k_{i}^{\left[s\right]}$ [21]. The resolution associated with each view is dictated by $\gamma_{v}$. $B_{\left(i,j\right)}^{\left[s\right]}={𝒜}_{\left(i,j\right)}^{\left[s\right]}-\gamma_{v}\frac{k_{i}^{\left[s\right]}k_{j}^{\left[s\right]}}{2m^{\left[s\right]}}$, where ${𝒜}_{\left(i,j\right)}^{\left[s\right]}$ is the adjacency matrix for view *s*. $J^{\left[sr\right]}$ represents the coupling between view *s* and *r*.

Where

- $k_{i}^{\left[s\right]}=\sum_{j}{𝒜}_{\left(i,j\right)}^{\left[s\right]}$: the degree (or weighted degree) of node *i* in view *s*.
- $2\mu^{\left[s\right]}=\sum_{ij}{𝒜}_{\left(i,j\right)}^{\left[s\right]}$: the total weight of all edges present in view *s*.
- $\gamma_{s}$: the resolution parameter associated with view *s*, which regulates the granularity of the detected communities (higher values of γ yield smaller communities).
- $C_{j}^{\left[sr\right]}$: the coupling strength connecting node *j* between view *s* and view *r*. In our proposed method, this value is determined by the structural consistency (Jaccard similarity) between the views.
- $c_{i},c_{j}$: the community assignments of node *i* and node *j*, respectively.
- [sr]: denotes the set of interactions between view *s* and view *r*.

**Figure 7:**
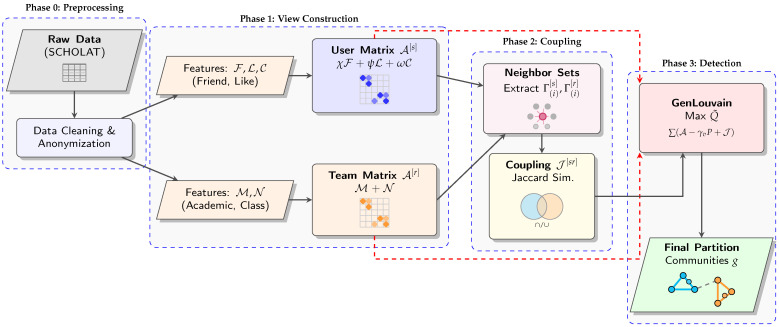
TaCD framework workflow: from raw data preprocessing to community detection through multi-view construction and cross-view coupling.

For clarity, Algorithm 2 summarizes the main procedure of the proposed method. The process flow of the dataset construction and TaCD algorithm is shown in Figure 7.
**Algorithm 2** The TaCD Algorithm**Input:** *n* (the number of nodes),${𝒜}^{\left[s\right]}$ (Adjacency matrix for User-view),${𝒜}^{\left[r\right]}$ (Adjacency matrix for Team-view),$\Gamma_{\left(i\right)}^{\left[s\right]},\Gamma_{\left(i\right)}^{\left[r\right]}$ for i∈[1,n] (Neighbor sets for both views),$\gamma_{v}$ (Resolution parameter).**Output:** The community assignment vector $g\in N^{n}$. 1:                   ▹ Step 1: Compute the cross-view coupling 2:Compute the diagonal coupling matrix $J^{\left[sr\right]}$ using Algorithm 1 with inputs $\Gamma_{\left(i\right)}^{\left[s\right]}$ and $\Gamma_{\left(i\right)}^{\left[r\right]}$. 3:                      ▹ Step 2: Define the objective function 4:Define the multi-view modularity objective function $\tilde{Q}$ using ${𝒜}^{\left[s\right]}$, ${𝒜}^{\left[r\right]}$, $J^{\left[sr\right]}$, and $\gamma_{v}$ as defined in Equation (10). 5:                     ▹ Step 3: Optimize the objective function 6:Optimize $\tilde{Q}$ using the Generalized Louvain algorithm [40] to find the final partition. 7:                ▹ Step 4: Obtain the final community assignments 8:Let $g\in N^{n}$ be the resulting community assignment vector, where $g_{i}$ is the community ID for node *i*. 9:**return** *g*

## 5. Experiments

### 5.1. Experimental Settings

In this set of experiments, we compare our approach with four existing state-of-the-art community detection techniques: AP (Affinity Propagation), NCut, Louvain, and VGAER. All experiments were conducted using a standard Personal Computer equipped with an Intel 3.4 GHz CPU and 16.0 GB RAM.

1. AP [41], which is a clustering algorithm based on “information transfer” between data points. AP algorithm does not need to determine the number of clusters before running the algorithm. The “examplars” searched by AP algorithm, e.g., clustering centroids, are the actual points on the dataset and represent each class;
2. NCut [42], which is a clustering method based on segmentation. The normalized cuts criterion measures both the total dissimilarity between the different groups as well as the total similarity within the groups. We show that an efficient computational technique based on a generalized eigenvalue problem can be used to optimize this criterion;
3. Louvain [2,43], which is an algorithm that optimizes modularity based on multi-level (round-robin heuristic). The modularity function is originally used to measure the quality of community detection algorithm results, and it is able to characterize the closeness of the communities found;
4. VGAER [44], which is a novel unsupervised community detection method based on Variational Graph AutoEncoder (VGAE). Unlike traditional deep learning methods that typically reconstruct the adjacency matrix, VGAER reconstructs the modularity matrix to capture high-order community structures effectively. It represents the state-of-the-art in graph neural network-based unsupervised community detection.

### 5.2. Evaluation Measures

Since on each testing network, ground-truth labeling is provided for evaluating the clustering accuracy, we compare three widely used evaluation measures: ACC [45], NMI [46], and ARI [47].

1. Accuracy (ACC) shows what percentage of the samples you have predicted are correct [48]. Given the node $V_{i}$, and $\pi_{i}$ is the assigned label of the node $V_{i}$, $\zeta_{i}$ is the real label of $V_{i}$ in the dataset. The ACC can be computed as
  (11) $$\begin{array}{c} ACC\left(\zeta ,\pi \right)=\frac{1}{n}\sum_{i=1}^{n}\delta \left(\zeta_{i},p_{\mathrm{map}}\left(\pi_{i}\right)\right) \end{array}$$
  where δ(x,y) is the Kronecker delta, which is the same as Equation (6). $p_{\mathrm{map}}\left(\pi_{i}\right)$ is the permutation mapping function that maps $\pi_{i}$ of node $V_{i}$ to the corresponding label in real community. *n* denotes the counts of nodes.
2. Normalized Mutual Information (NMI) is used for measuring the clustering accuracy based on the underlying class labels [49].
  Given a network G of size *n*, the clustering labels π of *c* clusters, and actual class labels ζ of $\hat{c}$ classes, a confusion matrix is formed first, where entry (i,j). $n_{i}^{\left(j\right)}$ gives the number of points in cluster *i* and class *j*. The NMI can be computed from the confusion matrix [42] as
  (12) $$\begin{array}{c} NMI\left(\zeta ,\pi \right)=\frac{2\sum_{l=1}^{c}\sum_{h=1}^{\hat{c}}\frac{n_{l}^{\left(h\right)}}{n}\log\frac{n_{l}^{\left(h\right)}n}{\sum_{i=1}^{c}n_{i}^{\left(h\right)}\sum_{i=1}^{\hat{c}}n_{l}^{\left(i\right)}}}{H\left(\zeta \right)+H\left(\pi \right)} \end{array}$$
  where $H\left(\zeta \right)=-\sum_{j=1}^{\hat{c}}\frac{n^{\left(j\right)}}{n}\log\frac{n^{\left(j\right)}}{n}$ and $H\left(\pi \right)=-\sum_{i=1}^{c}\frac{n_{i}}{n}\log\frac{n_{i}}{n}$ are the Shannon entropy of cluster labels *p* and ζ, respectively, with $n_{i}$ and $n^{\left(j\right)}$ denoting the number of points in cluster *i* and class *j*. A high NMI indicates the clustering and real labels match well. If π=ζ, $NMI\left(\zeta ,\pi \right)=1$. If π and ζ are completely different, $NMI\left(\zeta ,\pi \right)=0$.
3. Apart from ACC and NMI, in the comparison results, we also use Adjusted Rand Index (ARI) to validate the algorithm. ARI has become one of the most successful cluster validation indices, and it is recommended as the index of choice for measuring agreement between two partitions in clustering analysis with different numbers of clusters. The ARI can be computed as
  (13) $$\begin{array}{c} ARI\left(\zeta ,\pi \right)=\frac{2(ad-bc)}{\left(a+b)(b+d)+(a+c)(c+d\right)} \end{array}$$
  where *a* denotes the number of sample pairs in the same group of cluster ζ and class π; *b* denotes the same cluster in the original partition π, but the number of sample pairs in the cluster result ζ that are not in the same group; *c* denotes not in the same cluster π, but the number of sample pairs in the same in class ζ; *d* denotes the number of sample pairs in both cluster π and class ζ, which are not in the same group.

### 5.3. Parameter Analysis

In our proposed method, the resolution parameter $\gamma_{v}$ plays a crucial role, with a range of 0 to 2. We conducted a sensitivity analysis for this parameter to determine the optimal value for our experiments.

**Figure 8:**
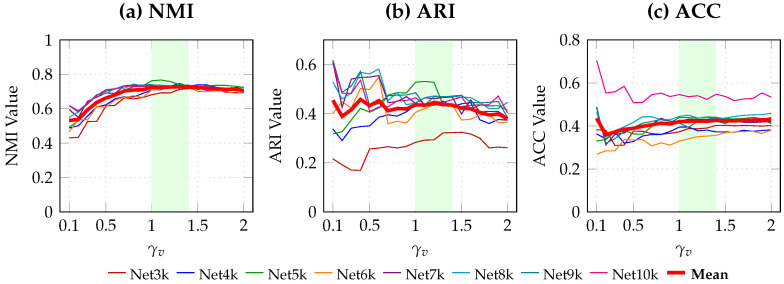
Parameter sensitivity analysis of $\gamma_{v}$ on NMI (**a**), ARI (**b**), and ACC (**c**). We employ distinct colors for individual networks (Net-3k to Net-10k) and a thick red line for the mean performance. The shared legend is placed at the bottom. The shaded green area highlights the robust parameter interval $\gamma_{v}\in \left[1.0,1.4\right]$.

As visually illustrated in Figure 8, the performance metrics (NMI, ARI, and ACC) across all eight networks exhibit a consistent trend. Crucially, a stable performance plateau is observed in the highlighted interval $\gamma_{v}\in \left[1.0,1.4\right]$. The mean performance curves (represented by the thick red lines) demonstrate that the algorithm has robust to small parameter variations within this region. Within this robust interval, while $\gamma_{v}=1.3$ yields a marginal peak in NMI, $\gamma_{v}=1.2$ demonstrates highly competitive stability and achieves a slightly stronger balance in ARI scores.

Considering this balance between near-optimal NMI and strong ARI performance, we set $\gamma_{v}=1.2$ as a robust and representative parameter to complete the community detection experiments with TaCD.

### 5.4. Complexity Analysis

The computational complexity of TaCD is determined by two main phases: the cross-view coupling construction (Algorithm 1) and the modularity optimization (Algorithm 2).

For the modularity optimization (Algorithm 2), we use the Generalized Louvain method. The complexity of the standard Louvain algorithm is known to be near-linear, often simplified to $O(M_{\mathrm{graph}}+n_{\mathrm{graph}})$ [43], where $n_{\mathrm{graph}}$ is the number of nodes and $M_{\mathrm{graph}}$ is the number of edges. In our multi-view model, the total number of nodes is *n*, and the total number of edges (intra-view plus inter-view couplings) is $M_{s}+M_{r}+n$. Thus, the optimization step has a complexity of $O\left(\left(M_{s}+M_{r}+n\right)+n\right)=O\left(M_{s}+M_{r}+n\right)$.

For the coupling construction (Algorithm 1), we compute the Jaccard similarity for all *n* nodes. This is sometimes mistakenly assumed to be an $O(n^{2})$ operation, which would involve computing a full similarity matrix. However, as shown in Algorithm 1, we only compute the *n* diagonal values (i=j). The complexity of computing the Jaccard similarity for a single node *i* is proportional to the size of its neighbor lists, $O(d_{i}^{\left[s\right]}+d_{i}^{\left[r\right]})$. Summing over all nodes, the total complexity for this phase is $\sum_{i=1}^{n}O\left(d_{i}^{\left[s\right]}+d_{i}^{\left[r\right]}\right)=O\left(M_{s}+M_{r}\right)$.

**Figure 9:**
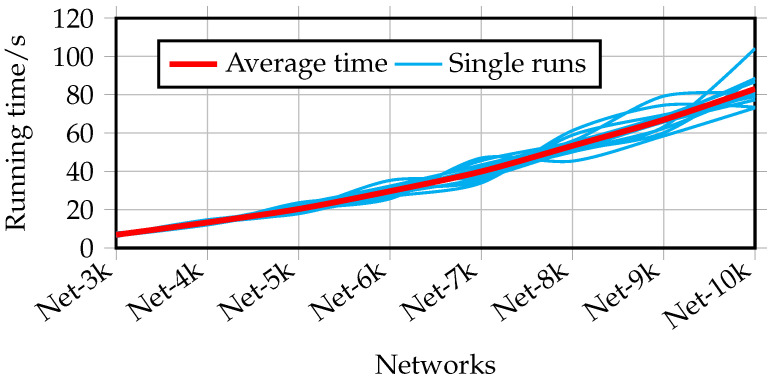
The running time of TaCD on different scale networks with γ∈[1,2].

Therefore, the total complexity of TaCD is $O\left(M_{s}+M_{r}\right)\;\left(\mathrm{Coupling}\right)+O\left(M_{s}+M_{r}+n\right)$ (Optimization). Letting $M=M_{s}+M_{r}$ be the total number of intra-view edges, the overall complexity simplifies to O(M+n). This near-linear complexity is highly scalable. The experimental running times shown in Figure 9 confirm this gentle, near-linear growth as the network size increases.

### 5.5. Comparison Results

With the proposed method, we utilize both the interaction information matrix and the team information matrix as inputs, serving as two distinct views for the network. In contrast, existing methods typically rely solely on the interaction matrix. The community structures detected by each method are compared against the ground-truth communities in the SCHOLAT dataset.

For the stochastic methods (NCut, Louvain, VGAER, and TaCD), we conducted ten independent runs for each network and reported the mean results to ensure statistical reliability. The deterministic algorithm AP was run once. The comprehensive results are presented in Table 4, where the best results are highlighted in bold and the second-best results are underlined.

**Figure 10:**
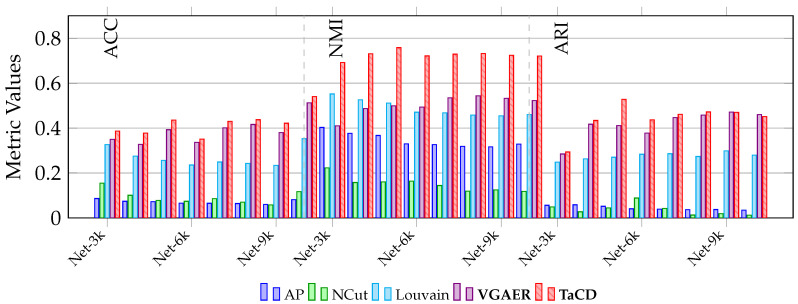
Comparison of ACC, NMI, and ARI metrics across eight networks.

As presented in Table 4 and Figure 10, we conducted a comprehensive evaluation including the advanced deep learning-based method, VGAER [44].

In terms of Accuracy (ACC), the proposed TaCD method consistently outperforms all baselines. It achieves a peak ACC of 0.5406 on Net-10k, which is notably higher than the deep learning-based VGAER (0.5122) and significantly surpasses traditional methods like AP (0.0811). This indicates that integrating team affiliations effectively corrects misclassified nodes in boundary regions.

Regarding Normalized Mutual Information (NMI), TaCD demonstrates its most significant advantage. By establishing a structural coupling between the user view and the team view, TaCD achieves NMI scores consistently in the range of 0.69–0.75. In comparison, VGAER, despite being a powerful GNN-based reconstruction method, fluctuates between 0.40 and 0.54. This validates that our multi-view modularity approach captures the global community structure more accurately than single-view reconstruction methods, which may struggle with the sparsity of social interaction data.

Interestingly, in the Adjusted Rand Index (ARI) metric, VGAER shows strong competitiveness, particularly on larger networks. As shown in Table 4, VGAER achieves ARI scores very close to TaCD, and even slightly surpasses TaCD on Net-9k (0.4711 vs. 0.4699) and Net-10k (0.4603 vs. 0.4512). This suggests that while TaCD is superior in global structure identification (NMI), VGAER is highly effective in pairwise classification decisions on large-scale graphs. Nevertheless, TaCD remains the best performing method overall, securing the highest scores across the vast majority of metrics and datasets.

### 5.6. Case Study and Visualization

**Figure 11:**
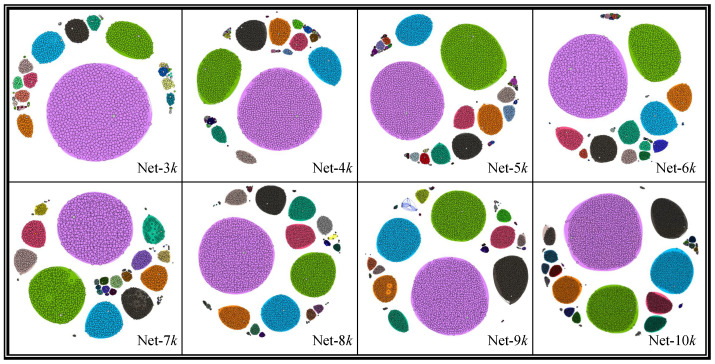
Results of case study and visualization: we can see that all nodes are clearly divided into several communities. There is a relatively large purple classification, whose name is *South China Normal University, Guangzhou, China*, that is the birthplace of SCHOLAT.

**Figure 12:**
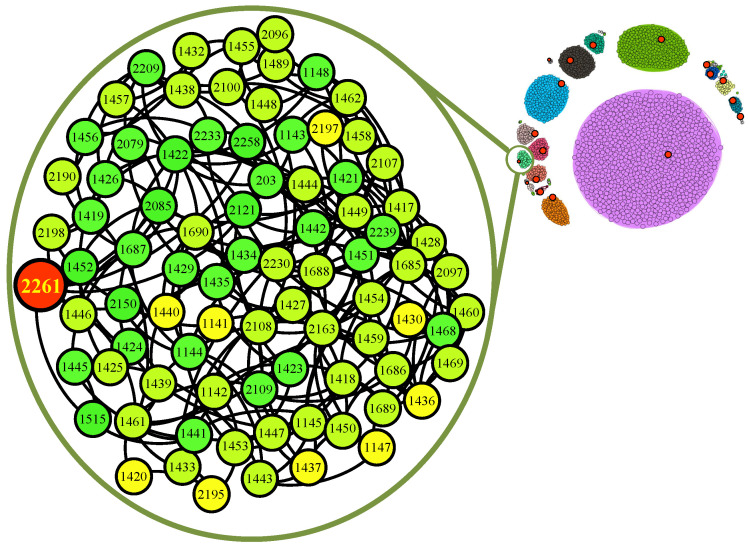
Community detection with TaCD on Net-3k: the big red node is the centroid of the community. In the enlarged view of a community, we can see that the centroid is node 2261. Otherwise, the community id is 33, and the name is *Guangdong Ocean University, China* in the real world.

The eight networks cluster results obtained by TaCD are shown in Figure 11, and the community information after processing with TaCD method is shown in Table 5. Next, we take a closer look at the experimental results. As Figure 12 shows, the big red node is the centroid of community, whose degree of edges is the highest. Through examples, what sets it apart from other community detection algorithms is that, TaCD is more sensitive to team information on the dataset networks and can fully utilize this information for more accurate community partitioning of nodes. Accurate segmentation can help us better develop recommendation algorithms on social networking platforms, thereby helping users create their social circles more quickly on the platform.

In addition, we find that among the eight clusters, there is a relatively large purple classification. By comparing the data in platform, we find that the classification is the greatest activity in SCHOLAT, while the name of largest class is *South China Normal University, Guangzhou, China*. In addition, the community is the birthplace of SCHOLAT.

### 5.7. Ablation Study

To verify that the performance gains are specifically driven by the team-aware modeling and not merely by parameter tuning or increased edge density, we conducted a comprehensive ablation study. We defined a baseline variant named TaCD-NoTeam, which utilizes only the Interaction Layer (User-view) by setting the cross-view coupling matrix to zero. We compared this baseline with the proposed TaCD-Full model, which fully integrates the Affiliation Layer (Team-view).

**Figure 13:**
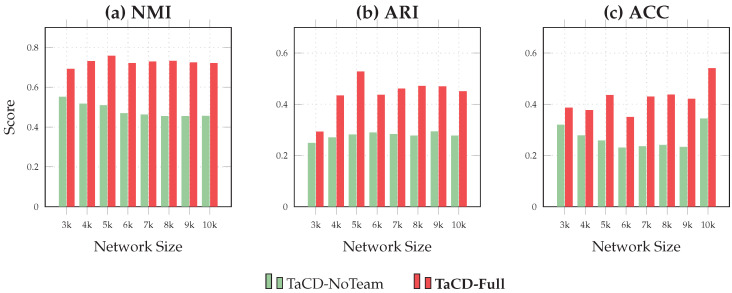
Ablation study results visualized as bar charts across all networks. The comparison highlights the performance gap between the baseline TaCD-NoTeam (green bars) and the proposed TaCD-Full (red bars) for NMI (**a**), ARI (**b**), and ACC (**c**). The consistent height difference across all datasets confirms the robustness of the team-aware strategy.

The comparative results across all eight networks (Net-3k to Net-10k) are visualized in Figure 13. In the figure, the green bars represent the performance of TaCD-NoTeam, while the red bars represent TaCD-Full. It is evident that the inclusion of team information yields a consistent and significant performance boost across all evaluation metrics (NMI, ARI, and ACC).

As illustrated in the figure, removing the team view results in a significant performance drop. This confirms that the explicit affiliation information contributes fundamentally to the detection accuracy.

## 6. Conclusions and Future Work

In this paper, we propose a novel method for community detection. The SCHOLAT dataset is combined with the characteristics of team attributes, and a multi-view approach is used to build a multi-layer community detection model based on team-aware named TaCD. The comparisons with other counterpart methods shows that the proposed method outperforms them. Furthermore, the dataset used for analysis is freely available to the research community to conduct further experiments on community detection. While our current implementation focuses on hard partitioning, which is essential for defining primary administrative units in organizations, we acknowledge that social communities often overlap. Future work will extend this multi-view framework to support overlapping community detection.

In the future, we plan to use more views beyond user-interactions view and team-relations view, and integrate more relationships to further enhance our method. For example, we can add time attributes, including interaction time, team creation time, and becoming friends time, which can form a new view. We can also apply the method to other datasets such as WebKB, SNAP_Pokec, DBLP, and so on, which consist of two or more types of relational data.

In addition, real networks always exhibit complex changes, and our methods are capable of conducting research on these dynamic networks. For example, our proposed multi-view approach can be used to solve community discovery problems in dynamic networks by treating networks before and after changes as different views.

## Figures and Tables

**Table 1:** The statistics of the SCHOLAT dataset.

Networks	Nodes	Edges	Communities	User ids
Net-3k	3000	689,480	157	1–3000
Net-4k	4000	992,387	162	1–4000
Net-5k	5000	1,385,964	178	1–5000
Net-6k	6000	2,462,527	178	1–6000
Net-7k	7000	3,107,667	186	1–7000
Net-8k	8000	4,061,459	197	1–8000
Net-9k	9000	5,009,882	208	1–9000
Net-10k	10,000	5,713,566	218	1–10,000

**Table 2:** Interaction among $u_{1},u_{2},u_{3},u_{4}$.

User Pair	Friendship	Like	Chat	${𝒜}_{\left(i,j\right)}^{\left[s\right]}$
$u_{1},u_{2}$	0	4	1	5
$u_{1},u_{3}$	1	7	0	9
$u_{1},u_{4}$	0	3	5	8
$u_{2},u_{3}$	0	9	6	15
$u_{2},u_{4}$	1	7	8	17
$u_{3},u_{4}$	0	0	0	0

**Table 3:** Common teams among $u_{1},u_{2},u_{3},u_{4}$.

User Pair	Common Team	Common Team Count	$A_{\left(i,j\right)}^{\left[r\right]}$
$u_{1},u_{2}$	c-F	1	1
$u_{1},u_{3}$	a-A	1	1
$u_{1},u_{4}$	a-D, c-G	2	2
$u_{2},u_{3}$	a-C, c-B, c-H	3	3
$u_{2},u_{4}$	a-E	1	1
$u_{3},u_{4}$	a-I	1	1

**Table 4:** Comparison of ACC, NMI, and ARI metrics across eight networks. The best results are highlighted in bold, and the second-best results are underlined.

	Networks	Algorithms				
		**AP**	**NCut**	**Louvain**	**VGAER**	**Ours (TaCD)**
ACC	Net-3k	0.0863	0.1548	0.3263	0.3500	**0.3869**
	Net-4k	0.0740	0.1008	0.2754	0.3278	**0.3776**
	Net-5k	0.0724	0.0776	0.2565	0.3928	**0.4360**
	Net-6k	0.0657	0.0741	0.2355	0.3367	**0.3507**
	Net-7k	0.0651	0.0857	0.2490	0.4011	**0.4302**
	Net-8k	0.0640	0.0701	0.2427	0.4160	**0.4375**
	Net-9k	0.0599	0.0579	0.2342	0.3802	**0.4217**
	Net-10k	0.0811	0.1166	0.3525	0.5122	**0.5406**
NMI	Net-3k	0.4027	0.2227	0.5522	0.4098	**0.6921**
	Net-4k	0.3767	0.1578	0.5256	0.4870	**0.7309**
	Net-5k	0.3671	0.1607	0.5118	0.4992	**0.7584**
	Net-6k	0.3299	0.1634	0.4709	0.4938	**0.7216**
	Net-7k	0.3268	0.1441	0.4683	0.5350	**0.7292**
	Net-8k	0.3183	0.1194	0.4576	0.5437	**0.7324**
	Net-9k	0.3165	0.1245	0.4547	0.5323	**0.7241**
	Net-10k	0.3288	0.1181	0.4602	0.5229	**0.7211**
ARI	Net-3k	0.0561	0.0489	0.2485	0.2846	**0.2935**
	Net-4k	0.0583	0.0273	0.2628	0.4172	**0.4340**
	Net-5k	0.0517	0.0442	0.2701	0.4108	**0.5279**
	Net-6k	0.0406	0.0884	0.2834	0.3777	**0.4369**
	Net-7k	0.0391	0.0424	0.2856	0.4467	**0.4610**
	Net-8k	0.0366	0.0129	0.2734	0.4579	**0.4720**
	Net-9k	0.0372	0.0187	0.2985	**0.4711**	0.4699
	Net-10k	0.0348	0.0122	0.2795	**0.4603**	0.4512

**Table 5:** The community information after processing with TaCD.

Networks	Nodes	Edges	Communities
Net-3k	3000	575,155	93
Net-4k	4000	897,233	107
Net-5k	5000	1,179,567	127
Net-6k	6000	1,888,510	121
Net-7k	7000	1,839,713	131
Net-8k	8000	2,693,429	139
Net-9k	9000	3,593,428	166
Net-10k	10,000	4,082,118	196

## Data Availability

The data are openly available in a public repository. The code is available at https://www.scholat.com/research/tacd, accessed on 13 November 2025.
